# Biochar Stimulated Actual Evapotranspiration and Wheat Productivity under Water Deficit Conditions in Sandy Soil Based on Non-Weighing Lysimeter

**DOI:** 10.3390/plants11233346

**Published:** 2022-12-02

**Authors:** Kholoud Z. Ghanem, Mostafa M. A. Hasham, Abdel-Nasser A. El-Sheshtawy, Rasha S. El-Serafy, Mohamed H. Sheta

**Affiliations:** 1 Department of Biological Science, Faculty of Science & Humanities College, Shaqra University, Riyadh 11961, Saudi Arabia; 2 Agronomy Department, Faculty of Agriculture, Al-Azhar University, Cairo 11651, Egypt; 3 Environment and Bio-Agriculture Department, Faculty of Agriculture, Al-Azhar University, Cairo 11651, Egypt; 4 Horticulture Department, Faculty of Agriculture, Tanta University, Tanta 31527, Egypt; 5 Soils and Water Department, Faculty of Agriculture, Al-Azhar University, Cairo 11651, Egypt

**Keywords:** water stress, wheat, biochar, actual evapotranspiration, water use efficiency

## Abstract

The major climate-related hazard to worldwide agricultural productivity is drought, which is becoming more common because of ongoing climate change, especially in the arid and semi-arid regions. Herein, we investigated the influence of biochar soil application at 0, (B1), 7.5 ha^−1^ (B2), and 15 t ha^−1^ (B3) on the productivity and drought-tolerance indices of wheat (*Triticum aestivum* L., cv. Sakha 93) grown in sandy soil under irrigation levels of 100 (I1), 80 (I2), and 60% (I3) of crop evapotranspiration (ETc), as well as soil properties based on non-weighing lysimeter units. Increasing water deficiency significantly decreased the actual evapotranspiration (ETa) values. A growing biochar rate caused a significant increase in ETa values, water use efficiency, and wheat productivity compared to the untreated control. Additionally, biochar supplementation revealed an improvement in soil quality as measured by the reduction in the bulk density and hydraulic conductivity with an increase in the total porosity and void ratio of the experimental soil. The correlation analysis exhibited a highly significant and positive correlation (0.98 **) between biological yield and grain yield traits. Therefore, it may be stated that these traits are the most significant components of the evaluated grain yield in wheat plants. The productivity of I1 plants was not significantly different and slightly higher than that of I2 plants. Therefore, it can be recommended that exposed wheat plants cultivated in sandy soil with I2 × B3 treatment significantly provide the highest yield while saving 20% of the irrigation water.

## 1. Introduction

Nowadays, water shortages all over the world are a critical issue that affects all the human life sectors and acts as a threat to food security. However, water requests for agriculture are growing worldwide to meet the increasing demand for food by the rapidly increasing population. The fast increase in the population percentage accompanied by economic and industrialization development [1] has altered freshwater ecosystems all over the world and led to a significant loss of biodiversity and an increase in freshwater utilization at unsustainable rates. In addition, the precipitation deficit and heat waves that hit almost all the world’s countries led to a significant decrease in the available water and lowered the soil water content, causing widespread stress on vegetation and threatening food security [2,3]. Altogether, we need extraordinary energy and water management measures to be applied in affected countries. Water stress negatively influences crop productivity and decreases crop yield. Effective management strategies may cause great production, accompanied by environmental and economic benefits [4]. Optimizing water use efficiency (WUE) is a beneficial strategy for improving crop productivity and yield production under sandy soil conditions.

Sandy soils are widely distributed worldwide and are often created when rocks like granite, limestone, and quartz are broken down or fragmented. They are identified by their poor structure, low levels of organic matter and nutritional properties, poor water holding capacity, and hydraulic properties. These soils limit plants’ roots from absorbing water, causing a reduction in crop productivity. Additionally, sandy soils have a very good drainage system. Organic soil supplements can help provide plants with an extra boost of nutrients by increasing the soil’s capacity to hold water and nutrients. Several studies have been conducted to improve soil–water relations using various applications, including biochar [4,5].

Biochar is the final product obtained by heating organic materials without oxygen [6]. It is produced from diverse sources [7], including forest debris, waste from food processing, crop residues, and manures, which are the common biochar feedstocks [7,8]. However, the features and functional ability of various kinds of biochar vary extensively [7] due to the differences in ash and organic installation of biomass materials [7,8]. Biochar can enhance soil fertility and carbon sequestration to minimize carbon emissions and alleviate climate change [9,10,11]. Charcoal is the dark carbon residue derived from plant substances by extracting water and other volatile constituents [12]. It produces an improvement in soil’s biological circumstances of soil microorganism quantity and quality [13]. These improvements can affect nutrient cycles and soil structure, resulting in differences in plant growth and productivity. Prakongkep et al. [5] found that biochar soil supplementation enhanced soil fertility and quality by enhancing water holding capacity, increasing cation exchange capacity, and retaining soil nutrients. Due to the high surface area and porous structure of biochar, its sustainable supplementation can positively influence the soil’s properties and water relations [14]. Recently, biochar has been identified as a strategy for enhancing WUE [15]. The long-term soil supplementation with biochar significantly enhanced water holding capacity [16] and soil water content [17]. Interestingly, biochar is described as a growth enhancer, as mustard productivity has been improved due to biochar application [18]. Similarly, biochar soil supplementation increased maize productivity by 50% under normal conditions [19]. Furthermore, biochar improved the oil yield of sunflower plants cultivated under water deficit conditions [20].

Wheat (*Triticum aestivum* L.) is one of the most important cereal crops and has a vital role in supplementing food security worldwide. Wheat, among other crops, feeds about one-fifth of the world’s population and its consumption has been increasing steadily [21]. To decrease the gap between wheat production and consumption all over the world, cultivation in the sandy soil is considered a horizontal expansion and a critical factor to increase the cultivated area with this important crop [22]. However, wheat growth and stability are threatened by drought and other disasters [23]. Drought on a global scale lowered wheat production by 20.6% between 1980 and 2015 [24]. Water deficiency is an abiotic stress affecting the growth and productivity of wheat at any growth stage (from germination to filling stages) [25,26]. Wheat production is mainly influenced by the irrigation rate and time at the different growth stages [27].

In this study, we are presumed to evaluate biochar soil application for mitigating water stress by using wheat (*Triticum aestivum* L., cv. Sakha 93) plants cultivated in sandy soil. It is predicted that the study findings will be efficient for formulating a management strategy for improving growth and wheat productivity under water stress when cultivated in non-weighing lysimeter units. Furthermore, the findings of this study might be beneficial for growing wheat in drought-affected regions worldwide.

## 2. Materials and Methods

### 2.1. Location and Materials’ Source

This experiment was conducted at the Experimental Farm of the Soils and Water Department, Agriculture Faculty, Al-Azhar University, Nasr City, Cairo, Egypt, (30°03′19.0″ N 31°19′10.0″ E), during the winter seasons of 2020/2021 and 2021/2022. Wheat (*Triticum aestivum* L., cv. Sakha 93) seeds were obtained from the Wheat Research Department, Field Crops Research Institute, Agricultural Research Center, Giza, Egypt. The climate conditions of the two seasons were collected by the Center Laboratory for Agricultural Climate (CLAC), Agricultural Research Center, Giza, Egypt, and presented as in Table 1.

### 2.2. The Non-Weighing Lysimeter

The non-weighing lysimeter was constructed using local substances. The tank is shaped like a circular plastic drum with a diameter of 50 cm and a height of 40 cm. The bottom of the tank was lightly tapered to facilitate water drainage with a 5 cm filter of sand and gravel. A drainage mechanism (funnel and hose) was provided to safely drain the percolated water from the lysimeter into a plastic bowl of 25 cm in diameter and 15 cm deep, which collects the drained water from the bottom of the lysimeter. The lysimeter units were filled with sandy soil collected from the surface layer (0–30 cm) of the Cairo-Alexandria Desert Road near Sadat City. Determined physical and chemical characteristics of the experimental soil (before planting) are shown in Table 2.

### 2.3. Biochar Preparation and Application

The biochar utilized in this study was made by slowly pyrolyzing *Eucalyptus* wood under an atmosphere of no oxygen at 450 °C [28]. Biochar was harvested using a stainless-steel mill, sieved through a 2 mm filter to remove large particles, dried by air, and then levelled using the equipment. Biochar was supplemented to each lysimeter before sowing and mixed thoroughly at rates of 0, 7.5, and 15 t ha^−1^. The chemical properties of used biochar are presented in Table 3.

### 2.4. Agronomical Crop and Treatments Applied

The seeds were sown on the 19 and 17 of November 2020 and 2021, respectively, in non-weighing lysimeter units at rate of 150 kg ha^−1^. This experiment was set up in a randomized complete block design in two factors with three replicates; the first factor was irrigation levels of 100 (I1), 80 (I2), and 60% (I3) of crop evapotranspiration (ETc); the second factor was biochar soil supplementation at rates of 0 (B1, control), 7.5 (B2), and 15 t ha^−1^ (B3). The seedlings were supplemented with the recommended dose of fertilizers and treated with irrigation levels 20 days after sowing manually.

### 2.5. Physicochemical Properties of the Soil

At the end of the growing season, three samples of the experimental soil from each treatment were collected for physical analysis. Soil particle size distribution was determined via the pipette method of Dewis and Freitas [29]. Pore size distribution was estimated by De-Leenher and De-Boodt [30]. Soil water suspension (1:2.5 *w*/*v*) was prepared to estimate soil pH according to Jackson [31] using a pH metre (Genway, UK), while ECe (dS m^−1^) was determined using an EC metre (Genway, UK) following the procedure of US Salinity Laboratory Staff [32]. The modified Walkey–Black method was assessed for organic carbon determination according to Allison [33]. The organic matter content was calculated by multiplying the organic carbon values by 1.72 [34]. Soil water retention was determined using the pressure plate procedure described by Klute [35] and James [36] at matric potentials of −0.33 and −15 bar for field capacity (FC%) and permanent wilting point (PWP%), respectively. The available water content (AW%) was computed as the difference between the soil water content at FC and PWP. Total porosity (%), bulk density (Mg m^−3^), and void ratio were determined according to Klute [35]. Saturated hydraulic conductivity was determined according to Klute and Direksen [37]. Soluble cations and anions as well as calcium carbonate were determined according to the methods of Jackson [31].

### 2.6. Plant Harvest

At the maturity stage (25 and 27 April for both seasons, respectively), 10 plants were manually harvested from each lysimeter unit, and plant height (cm), spike length (cm), grain no. spike^−1^ were estimated. All plants from each lysimeter unit were manually harvested and left to air dry for one week, and 1000-grain weight was determined. Spikes no. m^−2^ was estimated from all plants in each lysimeter unit and expressed to m^2^. The biological yield (grain and straw yield (t ha^−1^)) was estimated from all harvested plants in each lysimeter unit, and the standard grain moisture content of 13.5% was added to the yield computation. The harvest index (%) was calculated using the following equation:

(1) $$\mathrm{Harvest}\;\mathrm{index}\;(\%)=\frac{\mathrm{Grain}\;\mathrm{yield}\;\left(\mathrm{kg}\;{\mathrm{ha}}^{-1}\right)}{\mathrm{Biological}\;\mathrm{yield}\;\left(\mathrm{kg}\;{\mathrm{ha}}^{-1}\right)}\times 100$$

The crop index was calculated using the following equation:

(2) $$\mathrm{Crop}\;\mathrm{index}\;(\%)=\frac{\mathrm{Grain}\;\mathrm{yield}\;(\mathrm{kg}\;{\mathrm{ha}}^{-1})}{\mathrm{Straw}\;\mathrm{yield}\;(\mathrm{kg}\;{\mathrm{ha}}^{-1})}\times 100$$

### 2.7. Water Use Efficiency Determination

Water use efficiency (kg m^−3^) of the wheat plants was estimated, according to Giriappa [38], using the following equation:

(3) $$\mathrm{WUE}\;(\mathrm{kg}\;m^{-3})=\frac{Y}{\mathrm{ETa}}$$

where Y is the yield (kg ha ^−1^) and ETa is the actual crop evapotranspiration (m^3^ ha ^−1^).

### 2.8. Water Relations

Wheat plants were treated with three irrigation levels of 60, 80, and 100% of crop evapotranspiration manually via manual valves every 2 days. To calculate the amount of irrigation water applied (IWA) for each treatment, the reference evapotranspiration (ETo) was determined.

#### 2.8.1. Reference Evapotranspiration Computing

The reference evapotranspiration (ETo) was estimated according to Allen et al. [39] using the Penman–Monteith equation:

(4) $${\mathrm{ET}}_{o}\;(\mathrm{mm}\;{\mathrm{day}}^{-1})=\frac{0.408\Delta \left(R_{n}-G\right)+\gamma \frac{900}{T+273}u_{2}\left(e_{s}{-e}_{a}\right)}{\Delta +\gamma \left(1+{0.34u}_{2}\right)}$$

where Δ is the slope vapor pressure curve (kPa °C^−1^), Rn is the net radiation at the crop surface (MJ m^−2^ day^−1^), G is the soil heat flux density (MJ m^−2^ day^−1^), γ is the psychrometric constant (kPa °C^−1^), T is the mean daily air temperature at 2 m height (°C), U_2_ is the wind speed at 2 m height (m s^−1^), es is the saturation vapor pressure (kPa), e_a_ is the actual vapor pressure (kPa), and e_s_ − e_a_ is the saturation vapor pressure deficit (kPa). The average daily ETo was 4.13, 3.94, 2.70, 3.39, 5.13, and 6.55 mm day^−^^1^ for Nov., Dec., Jan., Feb., Mar., and Apr. of the first growing season, respectively, while 4.16, 3.97, 2.74, 3.45, 5.14, and 6.79 mm day^−^^1^ was the average daily ETo of the second season, respectively.

#### 2.8.2. Crop Evapotranspiration Estimation

Crop evapotranspiration (ETc) was measured according to Doorenbos and Pruitt [40] using the following equation and presented as mm day^−1^:

(5) $$\mathrm{ETc}\;(\mathrm{mm}\;{\mathrm{day}}^{-1})=\mathrm{ETo}\times \mathrm{Kc}$$

where ETo represents the reference evapotranspiration (mm day^−1^) and Kc represents the crop coefficient (dimensionless). The duration of the different crop growth stages was 20, 50, 60, and 30 days for initial, crop development, mid, and late stages, respectively, while the crop coefficients (Kc) of the initial, mid, and late stages were 0.7, 1.15, and 0.4, respectively, according to Allen et al. [39].

#### 2.8.3. Determination of Irrigation Water Applied 

The amount of irrigation water applied (IWA) was measured in the lysimeter area for each treatment at the end of each growing season. The irrigation water applied amount was calculated by using the following equation:

(6) $$\mathrm{IWA}\;\left(m^{3}{\mathrm{ha}}^{-1}\right)=\frac{\mathrm{ETc}\times A\times \mathrm{Ii}}{1000\times \left(1-\mathrm{LR}\right)}$$

where ETc represents the crop evapotranspiration (mm day^−1^), A represents the lysimeter area (m^2^), Ii represents the irrigation intervals (day), and LR represents leaching requirements (m^3^).

#### 2.8.4. Actual Evapotranspiration Estimation

The actual evapotranspiration (ETa) was calculated for each growth stage (initial stage (IS), development stage (DS), mid-stage (MS), and late stage (LS)) for both growing seasons. The rainfall received in the first season was 139.47 mm, while in the second season it was recorded as 76.32 mm. Actual evapotranspiration was calculated using the soil water balance method [39] and the following equation:

ETa (mm) = P + I − D ± ΔW(7)

where P represents the rainfall (mm), I represent the irrigation water applied to individual lysimeters (mm), D represents the deep percolation (mm), and ΔW represents the changes in soil water storage (mm).

### 2.9. Statistical Analysis

Experiments in the current study were conducted in a randomized complete block design. The experimental design included two factors: (1) irrigation levels (100, 80, and 60% of ETc) and (2) biochar soil supplementation (0, 7.5, and 15 t ha^−1^). The experiment was repeated twice in two successive seasons with three biological replicates for each treatment. The dataset of studied traits was collected and subjected to univariate and multi-variate statistical analysis. The normality test for data was preceded by the Shapiro–Wilk test. The combined analysis of variance (ANOVA) across two seasons for testing the significant differences within treatments was performed according to Gomez and Gomez [41] following homogeneity test of error variance using XLSTAT (Addinsoft, New York, NY, USA) statistical package. Tukey’s multiple range test was used to perform mean comparisons. Pooled data for traits across seasons, replications, irrigation levels, and biochar soil supplementation were used for correlation analysis and principal component analysis (PCA), and figures were plotted using Origin Pro 2021 (Origin Lab, Northampton, MA, USA).

## 3. Results

### 3.1. Combined ANOVA

The results found in Table 4 present the mean squares of the combined analysis for the main effects of the year, irrigation level (I), biochar concentrations (B), and their interaction for studied traits. The mean squares of the year showed non-significant differences in all studied traits. Meanwhile, the mean square of both irrigation and biochar as well as their interaction presented significant effects on all the studied traits.

### 3.2. Biochar Soil Supplementation Altered the Actual Evapotranspiration of Stressed Wheat Plants

**Figure 1:**
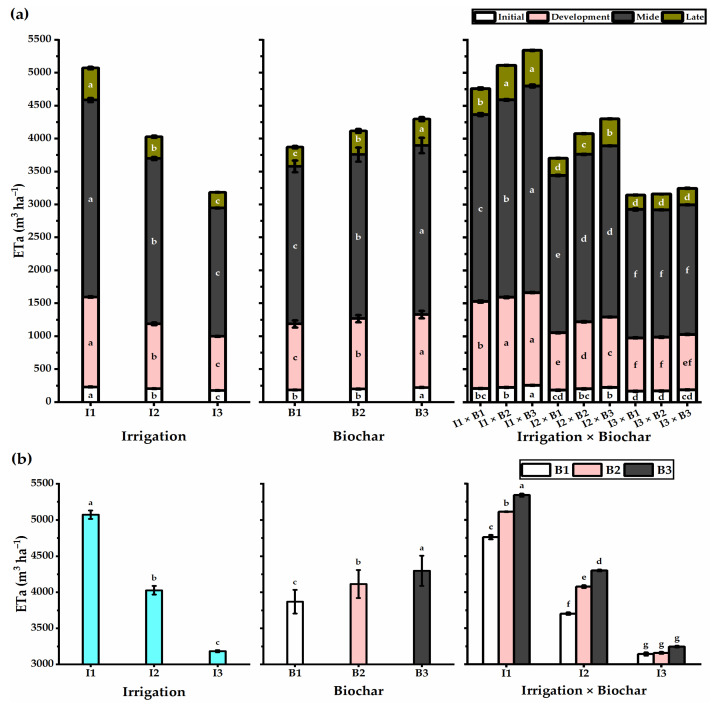
Actual evapotranspiration (ETa) for different growth stages (**a**) and total ETa (**b**) of the wheat plants affected by three irrigation levels of 100 (I1), 80 (I2), and 60% (I3) of crop evapotranspiration (ETc), and three levels of biochar soil supplementation 0 (B1), 7.5 (B2), and 15 t ha^−1^ (B3). Bars and error bars represent the means ± SE, respectively, of three biological replicates. Different letters reveal statistically significant differences among treatments.

The effects of different irrigation levels and biochar soil supplementation rates during the growth stages of wheat plants are presented in Figure 1. The higher ETa values were observed under 100% ETc irrigation and decreased with a reduction in the irrigation level during the growth stages. The highest ETa values were reported at the mid-stage (2990 m^3^ ha^−1^), followed by the development stage (1366 m^3^ ha^−1^), and the late stage (485 m^3^ ha^−1^). The lowest values of ETa were observed in the initial stage (228 m^3^ ha^−1^). In contrast to irrigation levels, increasing biochar levels led to an increase in ETa values, reaching the highest recorded at the B3 level (4296 m^3^ ha^−1^). By the means of the interaction, the I1 × B3 application significantly demonstrated the highest ETa values during different growth stages, as the mid-stage significantly obtained the highest ETa (3135 m^3^ ha^−1^) value, followed by the development stage (1408 m^3^ ha^−1^), while the lowest ETa value was gained by the initial growth stage (255 m^3^ ha^−1^).

The overall ETa values during wheat plant life showed great variations as affected by treatments applied, where decreasing the irrigation level to 60% ETc negatively affected the ETa values, causing a 37.2% decrease as compared with 100% ETc application. In contrast, the biochar applications significantly exhibited a 11% increase when wheat plants were exposed to B3 level. The plants that received the highest level of both irrigation and biochar (I1 × B3) significantly presented the highest ETa values, while I3 × B1 application caused the highest reduction in ETa value.

### 3.3. Biochar Soil Supplementation Stimulated the Yield Components and Biological Yield of Stressed Wheat Plants

**Figure 2:**
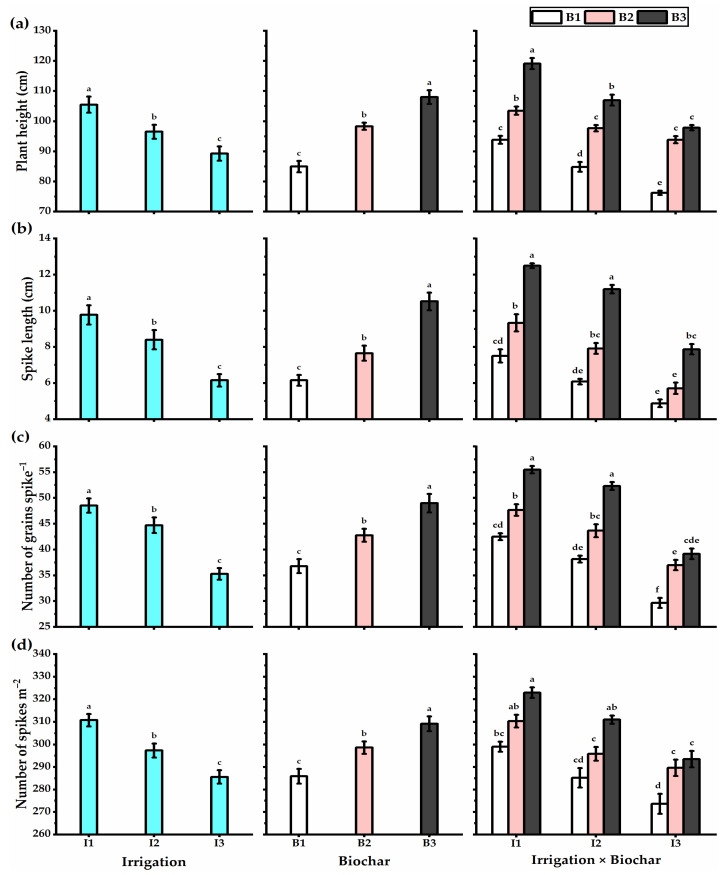
(**a**) Plant height (cm), (**b**) spike length (cm), (**c**) number of grains spike^−1^, and (**d**) number of spikes m^−2^ of the wheat plants affected by three irrigation levels of 100 (I1), 80 (I2), and 60% (I3) of crop evapotranspiration (ETc), and three levels of biochar soil supplementation, 0 (B_1_), 7.5 (B_2_), and 15 t ha^−^^1^ (B_3_). Bars and error bars represent the means ± SE, respectively, of three biological replicates. Different letters reveal statistically significant differences among treatments.

**Figure 3:**
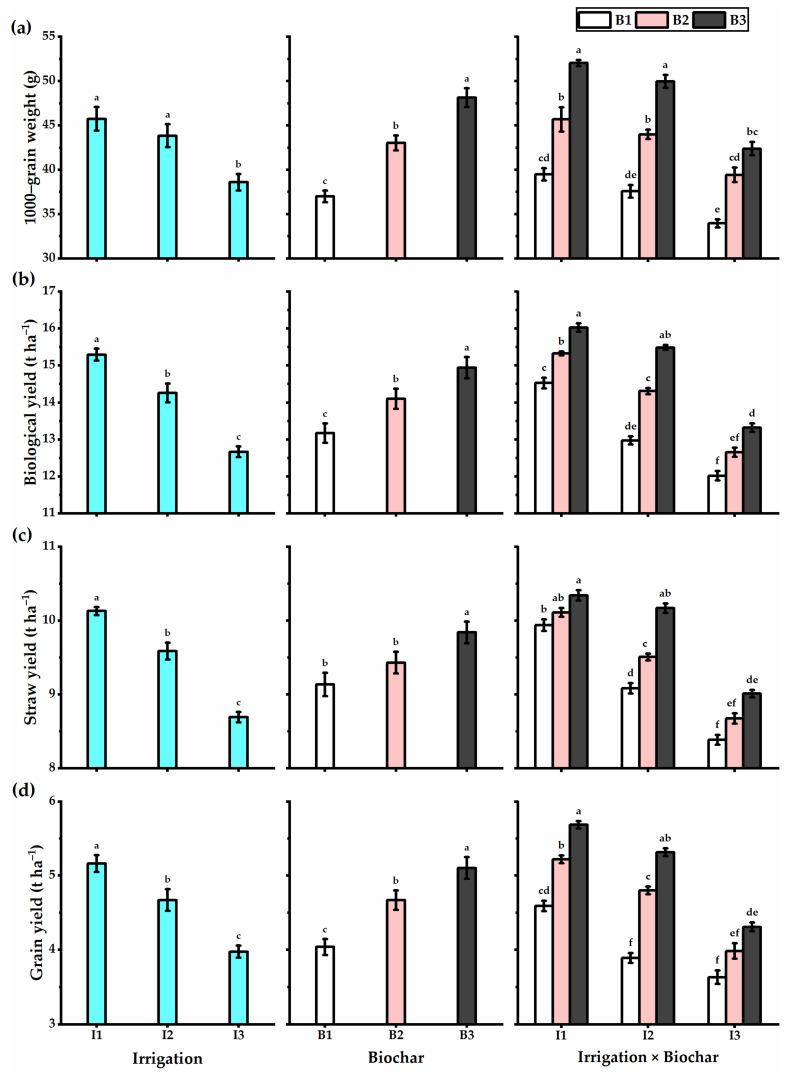
(**a**) 1000-grain weight (g), (**b**) biological yield (t ha^−1^), (**c**) straw yield (t ha^−1^), and (**d**) grain yield (t ha^−1^) of the wheat plants affected by three irrigation levels of 100 (I1), 80 (I2), and 60% (I3) of crop evapotranspiration (ETc), and three levels of biochar soil supplementation, 0 (B_1_), 7.5 (B_2_), and 15 t ha^−1^ (B_3_). Bars and error bars represent the means ± SE, respectively, of three biological replicates. Different letters reveal statistically significant differences among treatments.

**Figure 4:**
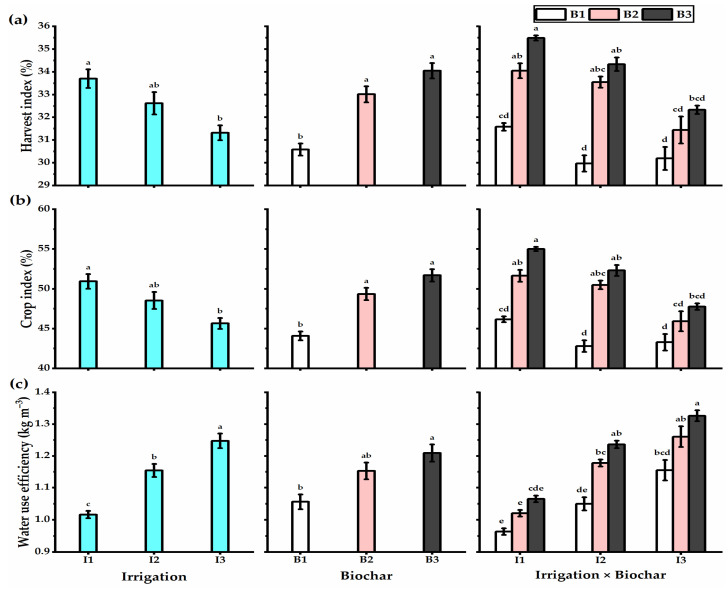
(**a**) Harvest index (%), (**b**) crop index (%), and (**c**) water use efficiency (kg m^−3^) of the wheat plants affected by three irrigation levels of 100 (I1), 80 (I2), and 60% (I3) of crop evapotranspiration (ETc), and three levels of biochar soil supplementation, 0 (B_1_), 7.5 (B_2_), and 15 t ha^−1^ (B_3_). Bars and error bars represent the means ± SE, respectively, of three biological replicates. Different letters reveal statistically significant differences among treatments.

Water deficiency negatively influenced the plant height, yield, and its components, including the spike length, grains no. spike^−1^, spikes no. m^−2^, 1000-grain weight, biological, straw, and grain yields, as well as the harvest and crop indices (Figure 2 and Figure 3). However, biochar soil addition significantly augmented the yield and yield components of wheat plants. Wheat plants treated with treated with I1 × B3 application had the highest plant height (119.2 cm), spike length (12.5 cm), grains no. spike^−1^ (55.5), spikes no. m^−2^ (323), 1000-grain weight (52 g), biological yield (16 t ha^−1^), straw yield (10.3 t ha^−1^), grain yield (5.7 t ha^−1^), harvest index (35.5%), and crop index (55%) (Figure 4). In this context, despite decreasing the irrigation level to I2 (80% ETc) caused a reduction in the yields obtained, biochar supplementation retained wheat productivity at the higher level, particularly by B3 application, which exhibited a non-significant difference with I1 × B3 treatment as gave spike length (11.2 cm), grains no. spike^−1^ (52.3), spikes no. m^−2^ (311), 1000-grain weight (50 g), biological yield (15.5 t ha^−1^), straw yield (10.2 t ha^−1^), grain yield (5.3 t ha^−1^), harvest index (34.3%), and crop index (52.3%). Otherwise, the wheat plants exposed to I3 × B3 application provided the lowest values in this respect. Concerning the WUE, wheat plants irrigated with I1 (100% ETc) exhibited the lowest WUE value, which increased with an augmentation of the irrigation level. Furthermore, biochar levels had the same trend as irrigation levels. The highest WUE was obtained by the wheat plants treated with I3 × B3 application (1.3 kg m^−3^).

### 3.4. Biochar Soil Supplementation Altered the Soil’s Physical Traits

**Figure 5:**
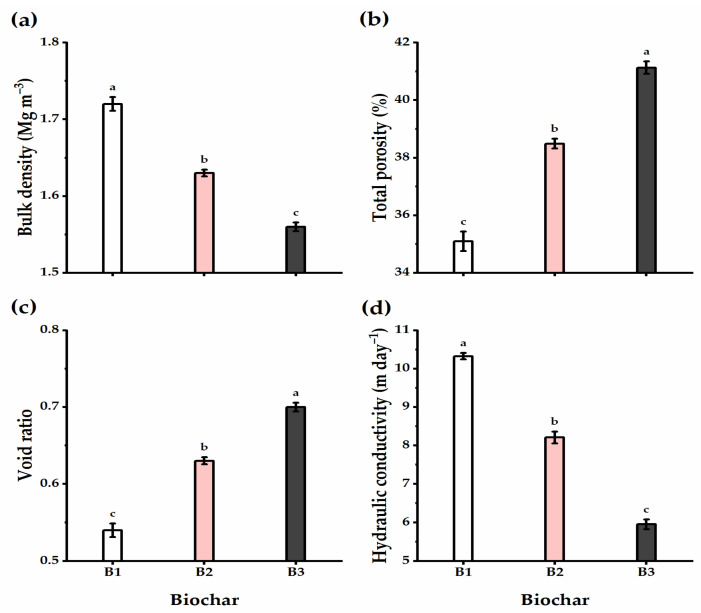
Effect of three levels of biochar soil supplementation (0 (B_1_), 7.5 (B_2_), and 15 t ha^−^^1^ (B_3_)) on the physical properties of soil cultivated with wheat at the harvest stage in sandy soil. (**a**) Bulk density (Mg m^−3^), (**b**) total porosity (%), (**c**) void ratio, and (**d**) hydraulic conductivity (m day^−1^). Bars and error bars represent the means ± SE, respectively, of three biological replicates. Different letters reveal statistically significant differences among treatments.

The effect of biochar soil addition on the physical properties of the experimental soil, including bulk density, total porosity, void ratio, and hydraulic conductivity, was evaluated (Figure 5). In brief, biochar application significantly decreased the bulk density and hydraulic conductivity of the experimental soil relative to untreated controls, reaching the lowest values at the high level of biochar application (B3) (1.56 Mg m^−3^ and 5.95 m day^−1^ for bulk density and hydraulic conductivity, respectively). The total porosity and void ratio traits of the experimental soil responded positively to the biochar soil supplementation level, as increasing the biochar level significantly increased their values, reaching the highest (41.13% and 0.7 e for total porosity and void ratio, respectively) when wheat plants received B3 application.

**Figure 6:**
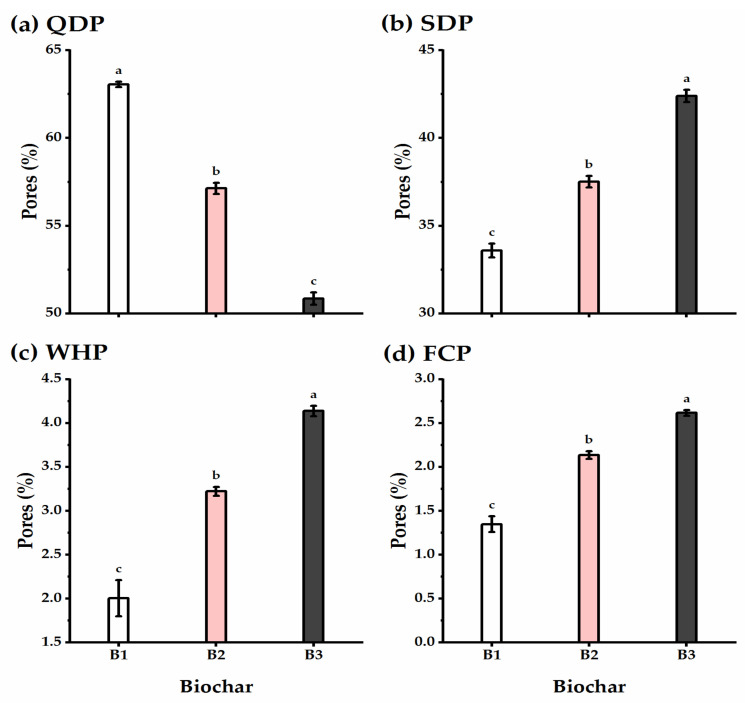
Effect of three levels of biochar soil supplementation (0 (B_1_), 7.5 (B_2_), and 15 t ha^−1^ (B_3_)) on the physical properties of soil cultivated with wheat at the harvest stage in sandy soil. (**a**) Quickly drainable pores (QDP,%), (**b**) slowly drainable pores (SDP,%), (**c**) water holding pores (WHP,%), and (**d**) fine capillary pores (FCP,%). Bars and error bars represent the means ± SE, respectively, of three biological replicates. Different letters reveal statistically significant differences among treatments.

The SDP, WHP, and FCP of pores showed an alternation influenced by biochar supplementation (Figure 6). Biochar application caused a gradual and significant increase in SDP, WHP, and FCP traits, recording the highest values following B3 application (42.39, 4.14, and 2.62% for SDP, WHP, and FCP, respectively). On the other hand, the QDP trait showed an adverse trend, as lysimeter units that received the highest rate of biochar showed the lowest QDP value, with a significant difference as compared with untreated units.

**Figure 7:**
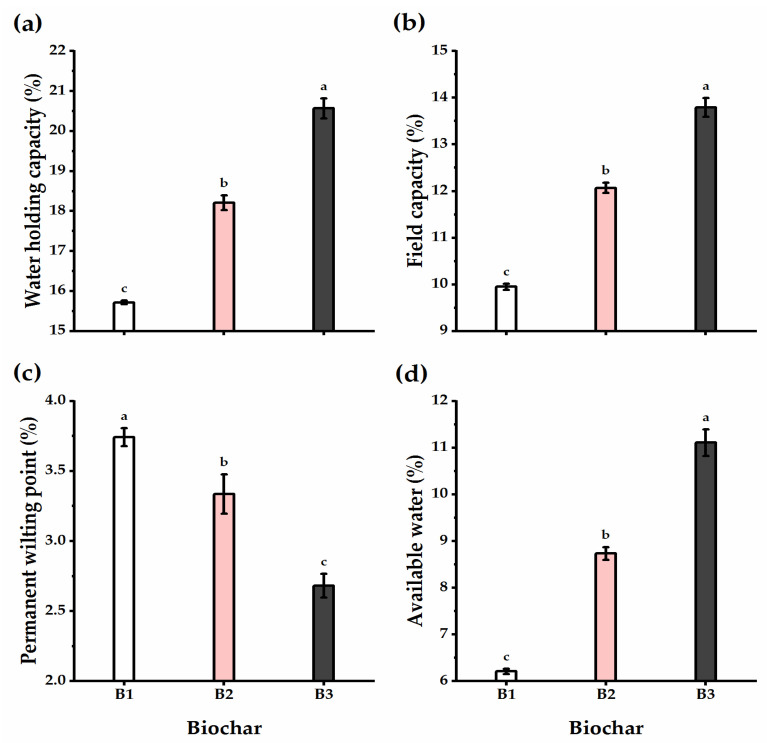
Effect of three levels of biochar soil supplementation (0 (B_1_), 7.5 (B_2_), and 15 t ha^−1^ (B_3_)) on the physical properties of soil cultivated with wheat at the harvest stage in sandy soil. (**a**) Water holding capacity (%), (**b**) field capacity (%), (**c**) permanent wilting point (%), and (**d**) available water (%). Bars and error bars represent the means ± SE, respectively, of three biological replicates. Different letters reveal statistically significant differences among treatments.

Under biochar addition, soil water relations were overall significantly enhanced as compared with untreated soils (Figure 7). The traits of water holding capacity, field capacity, and available water were greatly enhanced as affected by biochar application, particularly when increasing biochar level to the B3 treatment (the highest level), which recorded 20.57, 13.79, and 11.11% higher for water holding capacity, field capacity, and available water, respectively. Furthermore, when B2 and B3 were supplemented, the value of the permanent wilting point decreased by 10.9 and 28.4%, respectively, when compared to nontreated soils, though the differences were insignificant.

### 3.5. Correlation Analysis

**Figure 8:**
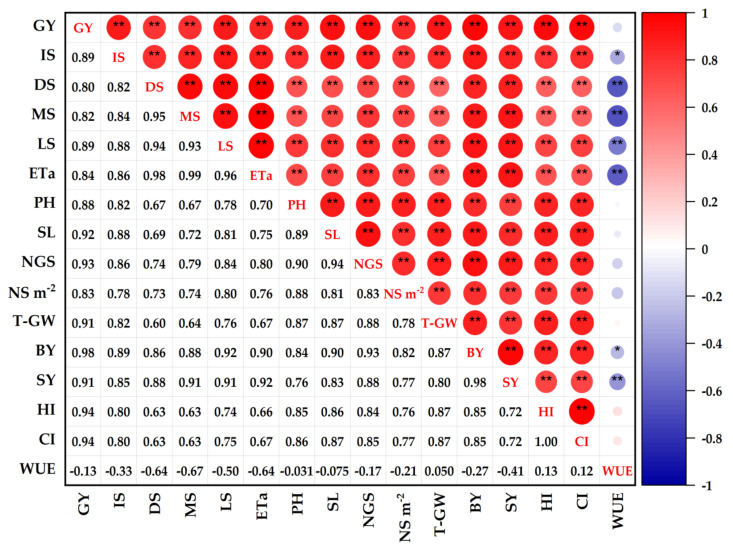
Matrix of Pearson’s correlation coefficients and correlation plots for studied traits. Pearson’s correlation coefficients are presented in the bottom−left half. The correlation plots are presented in the upper right half. The colours of the cells exhibit positive or negative correlations based on the colour scale. ** represent significance at 0.01. Grain yield (GY), ETa at initial stage (IS), ETa at development stage (DS), ETa at mid−stage (MS), ETa at late stage (LS), actual evapotranspiration (ETa), plant height (PH), spike length (SL), number of grains spike^−1^ (NGS), number of spikes m^−2^ (NS m^−2^), 1000−grain weight (T−GW), biological yield (BY), straw yield (SY), harvest index (HI), crop index (CI), water use efficiency (WUE).

Figure 8 depicts the direction of the relationship and the correlation coefficient values between the studied traits of wheat plants subjected to biochar soil supplementation and grown under different water deficiency levels. Based on the direction of the relationship, 89% of the correlation coefficients were positive and 11% were negative. By the means of the correlation coefficient values, the intensity of the relationship between the traits on the lower diagonal clearly indicates that 90% of the correlation coefficients have strong correlation coefficients and 10% have weak correlation coefficients. The grain yield (GY) trait presented a high positive and significant (*p* ≤ 0.01) correlation with all studied traits except WUE (−0.13), as GY exhibited high correlation with all yield component traits (SL, NGS, T–GW, BY, and SY). In addition, significant and high positive (*p* ≤ 0.01) correlations (r = 0.86 **,98 **,99 **, and 0.96 **, respectively) were demonstrated between ETa and IS, DS, MS, and LS stages, respectively. The WUE trait correlated negatively with almost studied traits, as it demonstrated a negative significant moderate correlation with DS (*r* = −0.64 **), MS (*r* = −0.67 **), LS (*r* = −0.50 **), and ETa (r = −0.64 **) traits. Meanwhile, weak positive correlation was demonstrated by HI and CI traits with WUE. Yield components showed a positive correlation, with the largest correlation coefficient of BY with GY (0.98 **). Due to the correlation coefficients between most of the studied traits being greater than 0.5, the correlation analysis showed a high degree of multidimensional overlapping relationships leading to fulfilling the requirements of the principal component analysis.

### 3.6. Principal Component Analysis

**Figure 9:**
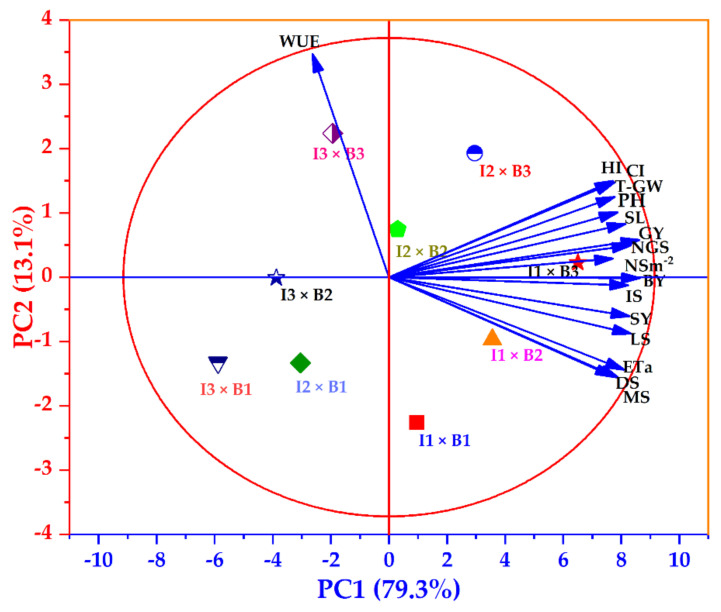
Biplot of the principal component analysis (PCA) of wheat plants subjected to biochar soil supplementation (0 (B_1_), 7.5 (B_2_), and 15 t ha^−1^ (B_3_)) under three levels of irrigation (100 (I1), 80 (I2), and 60% (I3) of ETC). The first and second principal components (PC1 and PC2) represented 92.4%. The vectors show the studied traits and the coloured shapes explained various treatments. Grain yield (GY), ETa at initial stage (IS), ETa at development stage (DS), ETa at mid−stage (MS), ETa at late stage (LS), actual evapotranspiration (ETa), plant height (PH), spike length (SL), number of grains spike^−1^ (NGS), number of spikes m^−2^ (NS m^−2^), 1000−grain weight (T−GW), biological yield (BY), straw yield (SY), harvest index (HI), crop index (CI), water use efficiency (WUE).

Principal component analysis (PCA) as a dimension reduction method was performed for all studied traits to determine the close relationship among different traits and treatments of the wheat plant as affected by biochar application under different irrigation levels on orthogonal dimensions (Figure 9 and Table S1). Two out of sixteen components (PC1 and PC2) were discovered with an eigenvalue larger than one (eigenvalue > 1), with values of 12.68 and 2.10, respectively. Meanwhile, the other PCs had eigenvalues less than one (eigenvalue < 1). In addition, components after the 10th have an eigenvalue close to or equal to zero. The variability for each component and cumulative (%) presented in Table S1 indicated that the two most important components were PC1 and PC2. PC1 shows the most variance in the dataset (79.26%), while the second principal component (PC2), which is orthogonal to the first component, explains 13.12% of the variance with a cumulative value of 92.38%. Other components, such as PC3 and PC4, which have variability values of 2.13 and 1.66, respectively, did not add effective values to the cumulative variance, since all components other than the first and second have weak variance values and eigenvalues of less than one. The contribution of variance and component loadings for the PC1 and PC2 components are shown in Table S1, which shows that all the traits have a contribution of variance on PC1 that is larger than that on PC2, except for DS, MS, Eta, HI, CI, and WUE. Excluding WUE, all traits have larger factor loading values on PC1 than on PC2. The score of factorial treatments on components showed that I1 × B3, I1 × B2, and I2 × B3 treatments had a high score in PC1 and were located near all traits except WUE (Figure 9), which was located near the I3 × B3 treatment, and had a greater score in PC2. Therefore, all traits reached their highest values with these treatments (I1 × B3, I1 × B2, and I2 × B3).

## 4. Discussion

### 4.1. Actual Evapotranspiration and Soil Characteristics

Water stress is a major threat to agricultural production, particularly in the arid and semi-arid ecosystems, causing an inhibition of plant growth and productivity. It is necessary to reduce environmental stresses to boost wheat output with an enhancement in soil quality. Recently, biochar soil addition has been reported as a technique for preserving agricultural sustainability, which has a significant potential for improving crop yield and reducing environmental stress [42]. Biochar supplementation has been shown to improve soil quality [43,44], increase water-fertilizer output [43], and decrease soil salinity [45].

Our results indicated that the highest ETa values were recorded by the I1 treatment (100%). The I1 application led to an increase in the rates of leaf transpiration and evaporation from the soil’s surface area. Similar findings were reported by El-Agrodi et al. [46], and by El-Nady and Shalaby [27]. Biochar soil addition showed an increase in ETa values, and the increase was more pronounced with an augmentation in the biochar level. The increase in ETa values following augmentation of the biochar rate might be attributed to the enhancement in the wheat yield, which led to an increase in water consumption. Similar results were reported by Andrenelli et al. [47], who indicated that biochar improved soil water retention properties, likely correlating with the inherent biochar retention potential and a more stable soil particle arrangement in the biochar pellets’ presence. The crop evapotranspiration was almost consistent in the mid-stage compared with different growth stages. At the late stage, the crop’s evapotranspiration showed a declining trend because of leaf senescence and completion of the grain formation, thereby filling and reducing transpiration. Likewise, Allen et al. [39] stated that crop water use decreased during the late stage, which they attributed to leaf growth cessation. The same author stated that nearly 100% of evapotranspiration comes from evaporation at the initial growth stage, and with the full coverage of the crop at the mid-growth stage, more than 90% of evapotranspiration comes from transpiration.

In this study, biochar soil addition enhanced soil properties as it decreased soil bulk density, and the reduction was more pronounced by increasing the biochar level. Blanco-Canqui, [48], Mendes et al. [49], and Toková et al. [50] reported that biochar application reduced soil bulk density, which may be attributed to (1) biochar properties, such as active surface area, particle size, and porosity, as well as soil properties; and (2) biochar’s ability to form soil aggregates in combination with soil particles, resulting in a decrease in soil bulk density. A gradual decrease in the soil bulk density with increasing biochar doses was also stated by Liu et al. [51]. Under stress conditions, biochar soil supplementation caused a slight enhancement in the soil’s water holding capacity with reducing irrigation frequency [16,52]. Similar results were observed by Laird [12] and Rani et al. [53], who stated that biochar soil addition increases available water, enhances affinity for soil nutrients, decreases leaching of nutrients to groundwater, and decreases the soil bulk density. Biochar increases soil water holding capacity (WHC), improves water availability to plants, promotes nutrient absorption, and boosts root development in the soil in both pot and field studies [54].

### 4.2. Wheat Productivity and Water Use Efficiency

Water stress might decrease roots’ growth, the amounts of nutrients in the root zone, and decrease assimilation translocation of the leaves, which negatively affects the photosynthesis rate, causing a decrease in the grain yield [55]. Wheat plants that received 80% ETc showed higher productivity and insignificantly differed from 100%-treated plants. These results are in line with the findings of Bakry et al. [56], and Moussa and Abdel-Maksoud [57]. A further reduction in water level applied resulted in a significant decrease in productivity and wheat components. Increasing biochar levels led to an improvement in the grain yield and wheat productivity. Biochar boosted the availability of essential elements (N, P, K, C, Ca, and Mg) in the soil as it acts as a nutrient source [58,59]. Furthermore, biochar absorbed elements and released them slowly, enhancing nutrient use efficiency. Cotton plants amended with biochar soil application exhibited higher physiological activity in their roots than untreated plants [43]. Biochar soil application enhanced stomatal conductance, leading to higher photosynthesis rates and total soluble sugar content in soybean plants relative to unamended soil [60]. Moreover, biochar soil addition caused an increase in N and chlorophyll content in wheat plants [61]. Mustard yield was increased following biochar application [18]. Similarly, maize production was improved following biochar soil application [19]. Under moderate and severe water deficiency, sunflower production improved following biochar application [20]. Higher concentrations of nutrients, protein, and lower reactive oxygen species in *Brassica chinensis* L. plants under stress conditions were observed [62]. Jeffery et al. [63] stated that biochar application in poor soils could increase crop yield by liming and fertilizing as well as improving the CEC of the soil. Agegnehu et al. [64] stated that wood biochar improved the WHC of soil, reduced N and P leaching, and increased the yields of peanut plants. The highest values of WUE were obtained by 60% plants, while the lowest values were obtained by 100% plants. The obtained findings are supported by Karam et al. [65], and El-Agrodi et al. [46], who reported that plants grown under water deficiency conditions had higher values than unstressed plants. Our results indicated that WUE was significantly affected by biochar supplementation as compared with the control treatment. Similarly, Abideen et al. [66], Ali et al. [67], and Wang et al. [68] observed that under drought stress treatment, the addition of biochar might lead to an increase in water use efficiency. On the PCA biplot, WUE matches the factorial treatment of 60% ETa with 15 t ha^−1^ biochar (Figure 9). Therefore, WUE does not increase with water shortage only but also by adding biochar; similar results were also previously reported by Telahigue et al. [69], and Aslam et al. [70]. Furthermore, biochar application significantly improved the WUE of wheat plants by improving soil physical properties and WHC [71]. Therefore, WUE is an important trait in PCA; this result is in line with Haider et al. [72], who reported that PCA explained that WUE is an important trait for wheat yield variations at critical growth stages. Tomato plants supplemented with biochar at 25 and 50 t ha^−1^ and cultivated under water deficit conditions exhibited an improvement in WUE [73]. Although the mechanism may be complicated, the impact of biochar on soil quality may have a significant impact on production. Therefore, it is suggested that higher crop yields in the biochar-amended trial may be linked to better soil quality and greater nutrient availability. Using all the studied traits, the first and second components were used on the biplot because their cumulative variance was 92.4%, while others possess eigenvalues lower than one, with very small variance. These findings were supported by Bjoklund, [74,75], who stated that when assessing the distinctness of the different PCs (eigenvalues), one should only take the distinct ones into consideration and ignore the rest. It is clearly presented in Figure 9 that I1 × B3 recorded the highest in most studied traits, but with non-significant differences compared to I2 × B3 values, and I2 × B3 had non-significant differences in comparison to I1 × B2. Based on the fact that saving water is the main goal of this study, saving 20% water is preferred through using the I2 × B3 treatment.

## 5. Conclusions

Biochar soil supplementation can be an effective strategy for improving the productivity of wheat plants growing in sandy soil under water deficiency conditions. Wheat productivity is not affected by decreasing the irrigation level to 80% ETc when biochar at the rate of 15 t ha^−1^ was supplemented, as it provided higher grains no. spike^−1^ (52.3), spikes no. m^−2^ (311), biological yield (15.5 t ha^−1^), straw yield (10.2 t ha^−1^), and grain yield (5.3 t ha^−1^). Therefore, 20% of the applied irrigation water can be preserved without detrimental yield when wheat plants are irrigated with 80% ETc and supplemented with 15 t ha^−1^ biochar.

## Figures and Tables

**Table 1:** Temperature (°C), wind speed (m s^−1^), relative humidity (%), average precipitation (mm day^−1^), and surface pressure (kPa) of experimental site during 2020/2021 and 2021/2022 winter growing seasons.

Month	Temperature (°C)		Wind Speed (m s^−1^)		Relative Humidity (%)	Average Precipitation (mm day^−1^)	Surface Pressure (kPa)
	Max	Min	Max	Min			
2020/2021							
November	24.64	13.66	5.15	1.87	63.38	0.36	100.10
December	22.57	10.47	5.08	1.97	60.63	0.02	100.14
January	21.52	8.33	5.82	2.34	59.04	0.07	100.26
February	21.78	8.29	5.26	2.05	61.52	0.72	100.26
March	23.27	9.20	6.11	2.09	62.38	3.36	100.06
April	29.43	11.71	6.54	2.63	50.20	0.13	99.98
2021/2022							
November	27.71	15.14	4.81	1.96	61.68	0.67	100.02
December	19.37	8.96	5.84	2.17	68.45	0.34	100.19
January	16.76	5.40	5.51	2.14	67.08	1.08	100.30
February	19.48	6.57	5.67	2.20	66.60	0.39	100.22
March	21.82	7.46	6.87	2.36	54.20	0.06	100.21
April	32.22	14.14	6.82	2.73	38.93	0.02	99.61

**Table 2:** Some physical and chemical properties of the experiment soil at sowing in both 2020/2021 and 2021/2022 winter growing seasons.

Property	2020/2021	2021/2022	Property	2020/2021	2021/2022
Particle size distribution			Field capacity (%)	9.98	9.92
Coarse sand (%)	51.36	51.36	Permanent wilting point (%)	3.73	3.75
Fine sand (%)	37.75	37.75	Available water (%)	6.25	6.17
Silt (%)	4.96	4.96	Water holding capacity (%)	15.71	15.73
Clay (%)	5.93	5.93	Bulk density (Mg m^−3^)	1.73	1.71
Textural class	Sandy	Sandy	Total porosity (%)	34.72	35.47
Ks (m day^−1^)	10.17	10.17	Void ratio	0.53	0.55
Pore size distribution			Soil pH (1:2.5) *	8.47	8.45
QDP (%)	63.03	63.09	ECe ** (dSm^−1^)	0.82	0.79
SDP (%)	33.65	33.52	Organic carbon (g kg^−1^)	2.59	2.58
WHP (%)	1.96	2.05	Organic matter (g kg^−1^)	4.45	4.44
FCP (%)	1.35	1.34	CaCO_3_ content (g kg^−1^)	13.71	13.74
Soluble cations			Soluble anions		
Ca^+ +^ (mmolc L^−1^)	1.86	2.02	CO_3_^−^ (mmolc L^−1^)	0.00	0.00
Mg^+ +^ (mmolc L^−1^)	3.25	3.57	HCO_3_^−^ (mmolc L^−1^)	5.20	4.98
Na^+^ (mmolc L^−1^)	2.17	1.64	Cl^−^ (mmolc L^−1^)	2.00	1.91
K^+^ (mmolc L^−1^)	0.90	0.70	SO_4_^−^ (mmolc L^−1^)	0.98	1.04

* 1:2.5 (*w*/*v*) soil water suspension, ** soil paste extract, Ks: saturated hydraulic conductivity, QDP: quickly drainable pores, SDP: slowly drainable pores, WHP: water holding pores, and FCP: fine capillary pores.

**Table 3:** Some chemical properties of biochar.

pH *	EC ** (dSm^−1^)	Organic Carbon (g kg^−1^)	Organic Matter (g kg^−1^)	CEC (cmolc kg^−1^)	C/N Ratio	Total N (g kg^−1^)	Total P (g kg^−1^)	Total K (g kg^−1^)
8.11	1.92	213.10	366.53	56.21	20.1:1	10.60	2.50	3.60

* 1:2.5 (*w*/*v*) biochar water suspension, ** biochar paste extract, and CEC: cation exchange capacity.

**Table 4:** Mean squares for the combined analysis of the soil traits as affected with biochar soil supple-mentation (orange colour). Mean squares of the combined analysis for the main effects of irri-gation, biochar, and the interaction for studied traits (green colour).

S.O.V	D.F	Bulk Density	Total Porosity	Void Ratio		Hydraulic Conductivity	
		(Mg m^−3^)	(%)			(m day^−1^)	
**Year (Y)**	1	0.0004	0.507	0.0004		0.192	
**R(Y)**	4	0.0001	0.159	0.0001		0.189	
**Biochar (B)**	2	0.0386 *	54.95 *	0.0386 *		28.68 **	
**B × Y**	2	0.0004	0.607	0.0004		0.015	
**Residual**	8	0.0003	0.421	0.0003		0.054	
**S.O.V**	**D.F**	**Pore Size Distribution (%)**					
		**QDP**	**SDP**	**WHP**		**FCP**	
**Year (Y)**	1	0.01	0.000	0.012		0.00002	
**R(Y)**	4	0.08	0.322	0.092		0.03733	
**Biochar (B)**	2	223.63 **	116.75 **	6.88 **		2.46 **	
**B × Y**	2	0.02	0.020	0.004		0.0003	
**Residual**	8	0.89	1.233	0.134		0.0213	
**S.O.V**	**D.F**	**WHC**	**Field Capacity**	**Permanent Wilting Point**		**Available Water**	
		**(%)**					
**Year (Y)**	1	0.009	0.001	0.0027		0.006	
**R(Y)**	4	0.158	0.092	0.1616		0.393	
**Biochar (B)**	2	35.32 **	22.20 **	1.72 **		36.05 **	
**B × Y**	2	0.002	0.003	0.0004		0.002	
**Residual**	8	0.289	0.166	0.0353		0.182	
**S.O.V**	**D.F**	**IS**	**DS**	**MS**	**LS**	**ETa**	
				**(m^3^ ha^−1^)**			
**Year (Y)**	1	0.22	53.59	1781.22	78.34	1693.5	
**R (Y)**	4	675.23	175.63	171.40	47.81	2048.0	
**Irrigation (I)**	2	13,345.7 **	1,396,194.8 **	4,887,211.4 **	286,161 **	16,093,538.7 **	
**Y × I**	2	17.61	8.25	567.81	6.08	795.7	
**Biochar (B)**	2	6236.8 **	50,872.7 **	139,386.2 **	53,893.7 **	823,830.4 **	
**Y × B**	2	50.49	262.34	423.07	80.37	1507.0	
**I × B**	4	340.52 **	12,572.3 **	33,686.9 **	9352.3 **	125,769.8 **	
**Y × I × B**	4	16.95	119.63	493.48	16.85	832.8	
**Residual**	32	98.44	421.31	1064.92	288.74	2101.7	
**S.O.V**	**D.F**	**PH**	**SL**	**NGS**	**NS m** **^−^** **^2^**	**T-GW**	
		**(cm)**	**(cm)**			**(g)**	
**Year (Y)**	1	228.17	0.01	0.30	1380.17	10.73	
**R (Y)**	4	12.15	1.96	13.74	71.37	4.89	
**Irrigation (I)**	2	1188.96 *	60.27 **	840.57 *	2854.6 *	246.86 *	
**Y × I**	2	16.89 *	0.28	8.91	58.50	4.04	
**Biochar (B)**	2	2412.80 **	88.85 **	672.30 **	2433.4 **	558.1 **	
**Y × B**	2	7.17	0.03	6.74	22.89	5.56	
**I × B**	4	54.30 *	2.13 **	19.77 *	53.35 *	9.21 *	
**Y × I × B**	4	5.06	0.04	1.60	7.39	1.13	
**Residual**	32	4.71	0.45	4.41	31.10	3.21	
**S.O.V**	**D.F**	**BY**	**SY**	**GY**	**HI**	**CI**	**WUE**
		**(t ha^−1^)**			**(%)**		**(kg m^−3^)**
**Year (Y)**	1	0.411	0.211	0.033	0.03	0.17	0.0033
**R (Y)**	4	0.101	0.029	0.060	1.41	6.82	0.0035
**Irrigation (I)**	2	31.582 **	9.484 **	6.459 **	25.68 **	125.9 **	0.243 **
**Y × I**	2	0.001	0.001	0.001	0.01	0.04	0.0001
**Biochar (B)**	2	14.161 **	2.253 **	5.198 **	57.17 **	273.8 **	0.108 **
**Y × B**	2	0.004	0.000	0.002	0.04	0.17	0.0005
**I × B**	4	0.640 **	0.187 **	0.232 **	2.87 *	14.64 **	0.0034 *
**Y × I × B**	4	0.002	0.002	0.005	0.19	0.90	0.0003
**Residual**	32	0.070	0.024	0.031	0.79	3.57	0.0026

* and **: statistically significant differences at *p*-value <0.05 and 0.01, respectively.

## Supplementary Materials

The following supporting information can be downloaded at: https://www.mdpi.com/article/10.3390/plants11233346/s1. Table S1: Eigenvalue, variability (%), and Cumulative (%) for thirteen components. Contribution of the traits (%) and component loadings for studied traits on first and second components.
