# Supplementary material for: Biochar Stimulated Actual Evapotranspiration and Wheat Productivity under Water Deficit Conditions in Sandy Soil Based on Non-Weighing Lysimeter
Source: Plants (Basel). 2022 Dec 2;11(23):3346. doi: 10.3390/plants11233346 (PMC9735446; doi:10.3390/plants11233346)
Supplement: Supplementary file 1 [file plants-11-03346-s001.zip › plants-1991424-supplementary.pdf]

plants

Article

Biochar Stimulated Actual Evapotranspiration and Wheat Productivity under Water Deficit Conditions in Sandy Soil Based on Non-Weighing Lysimeter

Kholoud Z. Ghanem [1], Mostafa M.A. Hasham [2], Abdel-Nasser A. El-Sheshtawy [3,*], Rasha S. El-Serafy [4,*] and Mohamed H. Sheta [5]

1 Department of Biological Science, Faculty of Science &Humanities College, Shaqra University, Riyadh 11961, Saudi Arabia
2 Agronomy Department, Faculty of Agriculture, Al-Azhar University, Cairo 11651, Egypt
3 Environment and Bio-Agriculture Department, Faculty of Agriculture, Al-Azhar University, Cairo 11651, Egypt
4 Horticulture Department, Faculty of Agriculture, Tanta University, Tanta 31527, Egypt
5 Soils and Water Department, Faculty of Agriculture, Al-Azhar University, Cairo 11651, Egypt
* Correspondence: abdel_nasser2007@azhar.edu.eg (A.-N.A.E.-S.); rasha.elserafi@agr.tanta.edu.eg (R.S.E.-S.)

Table S1. Eigenvalue, variability (%), and Cumulative (%) for thirteen components. Contribution of the traits (%) and component loadings for studied traits on first and second components.

Component	PC1	PC2	PC3	PC4	PC5	PC6	PC7	PC8	PC9	PC10	PC11	PC12	PC13
Eigenvalue	12.683	2.099	0.341	0.265	0.190	0.132	0.096	0.079	0.057	0.031	0.023	0.002	0.001
Variability (%)	79.26	13.12	2.13	1.66	1.19	0.83	0.60	0.49	0.36	0.19	0.14	0.02	0.01
Cumulative (%)	79.26	92.38	94.51	96.17	97.36	98.19	98.79	99.28	99.64	99.83	99.97	99.99	100.0

Traits	Contribution of the traits (%)		Component loadings	
	PC1	PC2	PC1	PC2
GY	7.531	1.169	0.977	0.157
IS	6.871	0.058	0.934	-0.035
DS	6.067	8.493	0.877	-0.422
MS	6.319	8.573	0.895	-0.424
LS	7.045	2.716	0.945	-0.239
ETa	6.630	7.285	0.917	-0.391
PH	6.276	3.573	0.892	0.274
SL	6.727	2.412	0.924	0.225
NGS	7.018	0.851	0.943	0.134
NS m^{-2}	6.034	0.291	0.875	0.078
T-GW	6.116	5.492	0.881	0.340
BY	7.581	0.001	0.981	-0.004
SY	6.951	1.294	0.939	-0.165
HI	6.031	7.885	0.875	0.407
CI	6.100	7.629	0.880	0.400
WUE	0.703	42.278	-0.298	0.942

Grain yield (GY), ETa at initial stage (IS), ETa at development stage (DS), ETa at mid stage (MS), ETa at late stage (LS), actual evapotranspiration (ETa), plant height (PH), spike length (SL), number of grains spike^{-1} (NGS), number of spikes

m^{-2} (NS m^{-2}), 1000−grain weight (T−GW), biological yield (BY), straw yield (SY), harvest index (HI), crop index (CI), water use efficiency (WUE).
